# Study of Clinical Outcome and Healthcare Modalities of COVID-19 Patients Treated With Remdesivir at a Tertiary Care Teaching Hospital

**DOI:** 10.7759/cureus.21535

**Published:** 2022-01-23

**Authors:** Tejas A Acharya, Krupal J Joshi, Divyesh D Patel, Shyam N Shah, Dimple S Mehta

**Affiliations:** 1 Department of Pharmacology, C. U. Shah Medical College and Hospital, Surendranagar, IND; 2 Department of Community and Family Medicine, All India Institute of Medical Sciences, Rajkot, IND; 3 Department of Anatomy, C. U. Shah Medical College and Hospital, Surendranagar, IND; 4 Department of Rheumatology and Clinical Immunology, Christian Medical College, Vellore, IND

**Keywords:** clinical outcome, health care modalities, coronavirus, covid-19, remdesivir

## Abstract

Background

Effective treatment for COVID-19 infection is still under evaluation. Remdesivir is an approved drug for COVID-19 treatment and major countries have released guidelines on the use of remdesivir. Still, many factors are under evaluation which can determine the future use of remdesivir.

Aim

To study the clinical outcome and healthcare modalities of COVID-19 patients treated with remdesivir.

Materials and methods

A retrospective study was conducted through the clinical records of patients admitted to the tertiary care hospital between August 2020 and December 2020. All the patients who were administered remdesivir intravenously as per standard protocol were included in the study. Data were analyzed for statistical association between health care modalities and patient characteristics.

Results

Among 166 patients included, the mean age of patient who received remdesivir was 57.51 ± 12.98 years (95% confidence interval (CI), 30-84). The mean duration of stay and duration of oxygen requirement were 12.80 ± 5.99 (95% CI, 5-30) and 9.41 ± 7.47 (95% CI, 0-39) days, respectively. A total of 12 (7.23%) required assisted ventilation and the cure rate was 89.76% (149/166). Out of 166 patients, 105 (63.25%) had comorbidities, among which hypertension and diabetes were the most common. Significantly >60 year age group had a higher duration of oxygen requirement (u=2,639.5, p=0.01), while ≤60 year age group had a higher cure rate (X^2^=4.23, p=0.03) and a higher requirement for assisted ventilation (X^2^=4.77, p=0.02). Differences and associations in the above-mentioned health care modalities were not statistically significant for gender and comorbidity except that non-comorbid had a higher cure rate (X^2^=3.97, p=0.04). The odds ratio of comorbidity and cure rate was 1.07, while the association of the number of comorbidities with the duration of stay (p=0.62) and duration of oxygen requirement (p=0.35) was not statistically significant.

Conclusion

In remdesivir-treated patients, age affects utilization of health care modalities. Female, non-comorbid, and younger patients have better clinical outcomes.

## Introduction

From the emergence of the COVID-19 pandemic in 2019-2020 till now 271,376,643 confirmed cases and 5,324,969 deaths have been reported by the World Health Organization (WHO) [1]. Effective treatment for COVID-19 infection is still under evaluation. Remdesivir was granted for emergency use authorization by the US FDA on May 1, 2020 [2]. Remdesivir, or GS-5734, is a prodrug of a nucleoside analog with direct antiviral activity against several single-stranded RNA viruses, including SARS-CoV and Middle East respiratory syndrome coronavirus (MERS-CoV) [3]. The first cell-based studies of remdesivir also showed antiviral activity against the novel SARS-CoV-2 [4,5]. Various pre-clinical and clinical trials have been conducted so far to suggest a possible role of remdesivir against the novel SARS-CoV2 [6,7]. Major countries have released guidelines on the use of remdesivir [8]. Although it has faced warnings against use by WHO [9], it can still be considered as one of the frontline treatments to fight against COVID-19. After facing two big waves, India and other countries in the world are still under threat of a third wave due to the emergence of new strains. Surveillance of drugs after marketing is as important as randomized clinical trials to have a constant eye on the effectiveness and safety of a drug [10]. Till now, many studies [7,11-13] have been conducted to infer the effectiveness of remdesivir, but very few studies [14,15] give an overview of the impact of various factors on remdesivir treatment. So, to have more access to factors that can modify remdesivir treatment outcome, this study was designed to study the clinical outcome and healthcare modalities of COVID-19 patients treated with remdesivir.

## Materials and methods

Ethical consideration

The study was carried out at a tertiary care teaching hospital after obtaining written permission from the Institutional Ethics Committee (Human Research).

Study design and setting

It was an observational cross-sectional study. A retrospective study was carried out by analyzing the clinical records of patients who were admitted to a government facility of a tertiary care teaching hospital between August 2020 and December 2020.

Inclusion criteria

All the patients who were administered remdesivir intravenously as per standard protocol (200 mg loading dose on day one followed by a 100 mg maintenance dose daily from day two to minimum day five) [14] by the treating physician were included in the study.

Exclusion criteria

Patients who were administered remdesivir but stopped for any reason before day five were excluded from the study. Cases of death or discharge against medical advice (DAMA) before five days were also excluded.

Methodology

Collected data were categorized according to gender, age, and comorbidity. Various health care modalities were compared for given categories. Duration of stay, duration of oxygen requirement, the requirement of assisted ventilation, and cure rate were considered as health care modalities. Requirement of assisted ventilation was considered as a requirement for hi-flow nasal cannula or bipap or invasive ventilation during the hospital stay of a patient, irrespective of the duration of requirement. Patients who required simple oxygen masks or non-rebreather masks (NRBMs) were not considered in the requirement of assisted ventilation category. For the cure rate, patients who were either clinically cured or given discharge on request were considered as cured, while death, referred and DAMA patients were considered uncured.

Statistical analysis

Collected data were compiled and analyzed using Microsoft Excel 2010 (Microsoft Corporation, Redmond, Washington, USA). Descriptive statistics (mean ± standard deviation with 95% confidence interval (CI)) was calculated for quantitative data related to age, gender, duration of stay, duration of oxygen requirement, and total population. The Shapiro-Wilk and Kolmogorov-Smirnov tests were the first normality tests. After obtaining result from these tests depending upon results non-parametric test (Mann-Whitney U test) or parametric test (Student's T test) was done for the duration of stay and duration of oxygen requirement. The comparison of data having more than two groups (number of comorbidity vs. duration of stay/duration of oxygen requirement) was done by using the Kruskal-Wallis test (after normality test). A chi-square test was used for the analysis of qualitative data. The odds ratio was calculated to find the association between cure rate and comorbidity. For all, p<0.05 was kept as the statistically significant level.

## Results

Patient characteristics

Out of 185 patients' clinical records analyzed, 166 patients were included in the study. Out of 19 excluded patients, five were referred, five were discharged, and nine did not satisfy study setting or inclusion criteria. The mean age of patients admitted was 57.51 ± 12.98 years (95% CI, 30-84). The mean duration of stay and mean duration of oxygen requirement were 12.80 ± 5.99 (95% CI, 5-30) days and 9.41 ± 7.47 (95% CI, 0-39) days, respectively. A total of 12 (7.23%) required assisted ventilation and the cure rate was 89.76% (149/166). Out of 166 patients, 88 (53.01%) were ≤60 years and 78 (46.99%) were >60 years of age. Among hospitalized patients who received remdesivir, 115 (69.28%) were male and 51 (30.72%) were female. Most of the patients had ≥two complaints simultaneously. The most common presenting complaint was breathlessness (n=81), followed by cough (n=80), fever (n=79) and weakness (n=38). Comorbidity assessment shows that out of 166 patients, 105 (63.25%) had comorbidity, while 61 (36.75%) were not having any comorbid conditions. Hypertension (n=71) was the most common comorbidity, followed by diabetes (n=70), ischemic heart disease (n=11), hypothyroidism (n=8), bronchial asthma (n=2), tuberculosis (n=1), and epilepsy (n=1). For further analysis, data were categorized according to gender, age, and comorbidity.

Gender-wise analysis of remdesivir-treated patients

Gender-wise description with age, presenting complaint, and comorbidity is summarized in Table 1. Remarkable findings were that ≥two presenting complaints and ≥two comorbidities were both higher in females.

**Table 1:** Gender-wise patient characteristics.

Patient characteristics	Presentation	All (n=166)	Male (n=115)	Female (n=51)
Age (years)	Mean (95% CI)	57.51 ± 12.98 (30-84)	59.18 ± 14.14 (30-90)	59.37 ± 11.98 (23-80)
	≤60	88 (53.01)	61 (53.04)	27 (52.94)
	>60	78 (46.99)	54 (46.96)	24 (47.06)
Presenting complaint	≥2	104 (62.65)	67 (58.26)	37 (72.55)
	1	60 (34.14)	46 (40)	14 (27.45)
	0	2 (1.21)	2 (1.74)	0 (0)
Comorbidity	None	61 (36.75)	51 (44.35)	10 (19.61)
	1	51 (30.72)	37 (32.17)	14 (25.45)
	≥2	54 (32.53)	27 (23.48)	27 (52.94)
	Hypertension	71 (100)	38 (53.52)	33 (46.48)
	Diabetes	70 (100)	40 (57.14)	30 (42.86)

Statistical analysis was done for the health care modalities that are shown in Table 2. As it is evident from Table 2, females had more duration of stay and more duration of oxygen requirement. Results obtained from normality tests done by Shapiro-Wilk (p=0.00 for both) and Kolmogorov-Smirnov (p=0.05 for both) were significant. It concluded that data did not follow normal distribution. To establish a statistical difference after the normality test, a Mann-Whitney U test, was done which showed no significant difference in duration of stay (u=2,639.5, p=0.30) and duration of oxygen requirement (u=2,629.0, p=0.29). As shown in Table 2, more number of male patients required assisted ventilation compared to females. An association between two variables named "required assisted ventilation" and "not required assisted ventilation" with females and males was studied. A Yates chi-square test was used to check this association (p=0.90). It indicates that there is no significant association between genders for requirement of assisted ventilation (X^2^=0.02). Cure rate was apparently higher in females as compared to males, but this association was statistically not significant (X^2^=0.01, p=0.90).

**Table 2:** Gender association with health care modalities. *u value denotes value of Mann-Whitney U test, **p<0.05 is kept at a statistically significant level, †duration is in days and mean is with 95% CI.

Healthcare modalities	All (n=166)	Male (n=115)	Female (n=51)	Statistical test	p^**^ value
Duration of stay^†^	12.80 ± 5.99 (5-30)	12.18 ± 5.84 (4-30)	12.80 ± 5.99 (5-30)	u^*^=2,639.5	p=0.30
Duration of oxygen requirement^†^	9.41 ± 7.47 (0-39)	8.35 ± 6.03 (0-27)	9.41 ± 7.47 (0-39)	u^*^=2,629.0	p=0.29
Requirement of assisted Ventilation	12	8	4	X^2^=0.02	p=0.90
Cure rate	149 (89.76)	103 (89.57)	46 (90.20)	X^2^=0.01	p=0.90

Age-wise analysis of remdesivir-treated patients

Age-wise analysis for gender, presenting complaint, and comorbidity is illustrated in Table 3. Noteworthy findings are that ≥two presenting complaints were higher in the ≤60 years age group, while ≥two comorbidities were higher in the >60 years age group. The rate of diabetes was surprisingly higher in the ≤60 years age group.

**Table 3:** Age-wise patient characteristics.

Patient characteristics	Presentation	All (n=166)	Age ≤60 (n=88)	Age >60 (n=78)
Gender	Male	115 (69.28)	61 (69.32)	54 (69.23)
	Female	51 (30.72)	27 (30.68)	24 (30.77)
Presenting complaint	≥2	104 (62.65)	60 (68.18)	44 (56.41)
	1	60 (36.15)	26 (29.55)	34 (43.59)
	0	2 (1.20)	2 (2.27)	0 (0)
Comorbidity	None	61 (36.75)	36 (40.91)	25 (32.05)
	1	51 (30.72)	27 (30.68)	24 (30.77)
	≥2	54 (32.53)	25 (28.41)	29 (37.18)
	Hypertension	71 (100)	18 (25.35)	53 (74.65)
	Diabetes	70 (100)	39 (55.71)	31 (44.29)

In Table 4, age-associated with health care modalities is conveyed. It is apparent that the duration of stay and the duration of oxygen requirement were higher in >60 years age group. Normality tests done by the Shapiro-Wilk test (duration of oxygen requirement p=0.00, duration of stay p=0.02) and Kolmogorov-Smirnov (duration of oxygen requirement p=0.00, duration of stay p=0.00) indicated that these data were not normally distributed. So, the Mann-Whitney U test was done, which revealed a significant difference in the duration of oxygen requirement (u=2,639.5, p=0.01), while the difference in the duration of stay was partially significant (u=2,810.0, p=0.06). Table 4 shows that age group ≤60 required more assisted ventilation. To check this, a chi-square test was done, which showed a significant association (X^2^=4.77, p=0.02). The cure rate was significantly higher in the ≤60 year age group (X^2^=4.23, p=0.03).

**Table 4:** Age association with health care modalities. *u value denotes value of the Mann-Whitney U test, **p<0.05 is kept at a statistically significant level, and †duration is in days and mean is with 95% CI.

Healthcare modalities	All (n=166)	Age ≤60 (n=88)	Age >60 (n=78)	Statistical test	p** value
Duration of stay^†^	12.80 ± 5.99 (5-30)	12.35 ± 5.75 (4-30)	13.12 ± 6.13 (5-32)	u*=2,810.0	p=0.06
Duration of oxygen requirement^†^	9.41 ± 7.47 (0-39)	8.86 ± 7.43 (0-39)	9.57 ± 5.45 (0-25)	u*=2,639.5	p=0.01
Requirement of assisted ventilation	12	10	2	X^2^=4.77	p=0.02
Cure rate	149 (89.76)	83 (94.31)	66 (84.61)	X^2^=4.23	p=0.03

Comorbidity-wise analysis of remdesivir-treated patients

Comorbidity-wise analysis according to age, presenting complaint, and gender is elucidated in Table 5. Highlighting findings are that non-comorbid patients had higher rate of ≥two presenting complaint and comorbid group had more male patients.

**Table 5:** Comorbidity-wise patient characteristics.

Patient characteristics	Presentation	All (n=166)	Comorbid (n=105)	Non-comorbid (n=61)
Age (years)	Mean (95% CI)	57.51 ± 12.98 (30-84)	60.41 ± 10.92 (37-90)	54.57 ± 14.87 (23-79)
	≤60	87 (52.41)	52 (49.52)	35 (57.38)
	>60	79 (47.59)	53 (50.48)	26 (42.62)
Presenting complaint	≥2	104 (62.65)	64 (60.95)	40 (65.57)
	1	60 (36.14)	41 (39.05)	19 (31.15)
	0	2 (1.21)	0 (0)	2 (3.28)
Gender	Male	115 (69.28)	64 (60.95)	51 (83.61)
	Female	51 (30.72)	41 (39.05)	10 (16.39)

The association of comorbidity with health care modalities is shown in Table 6. The duration of stay and duration of oxygen requirement were higher in patients in the comorbidity group. Normality tests done by the Shapiro-Wilk test (p=0.00 for both) and Kolmogorov-Smirnov (p=0.00 for both) showed that these data were not normally distributed. To establish the difference, after the normality test, the Mann-Whitney U test was done, which specified no significant difference in the duration of stay (u=2,900.5, p=0.31) and the duration of oxygen requirement (u=2,885.0, p=0.29). Table 6 also shows that comorbid patients required more assisted ventilation. This association was tested by the Yates chi-square test, which showed no significant association between comorbidity and the requirement of assisted ventilation (X^2^=0.42, p=0.51). The cure rate was significantly higher in non-comorbid patients (X^2^=3.97, p=0.04). The odds ratio for this association was 1.07 (95% CI), which advocates a strong association between exposures to comorbidity and cure rate.

**Table 6:** Comorbidity association with health care modalities. *u value denotes value of Mann-Whitney U test, **p<0.05 is kept at a statistically significant level, †duration is in days and mean is with 95% CI, OR: odds ratio.

Healthcare modalities	All (n=166)	Comorbid (n=105)	Non-comorbid (n=61)	Statistical test	p** value
Duration of stay^†^	12.80 (5-30)	13.02 (4-30)	11.76 (5-32)	u*=2,900.5	p=0.31
Duration of oxygen requirement^†^	9.41 (0-39)	9.73 (0-39)	8.18 (0-27)	u*=2,885.0	p=0.29
Requirement of assisted ventilation	12	9	3	X^2^=0.42	p=0.51
Cure rate	149 (89.76)	98 (93.33)	51 (83.61)	X^2^=3.97, OR=1.07 (95% CI)	p=0.04

**Figure 1:**
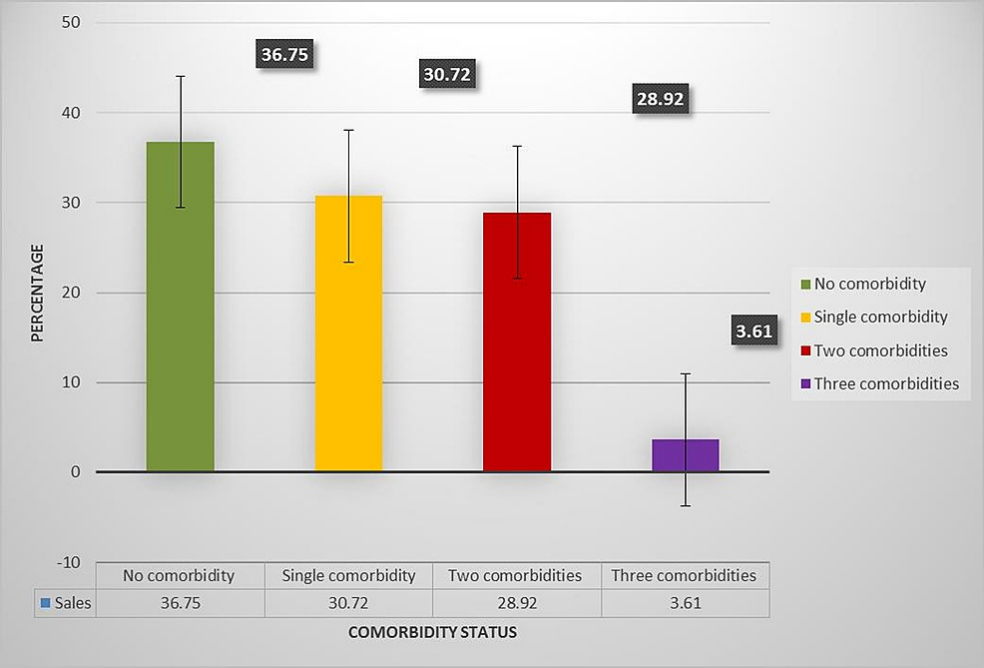
Percentage of patients according to the number of comorbidities.

Analysis according to the number of comorbidities is depicted in Figure 1. As comorbidity is most important prognostic factor further analysis was done to check the association of the number of comorbidities (zero, single, two, three) with the duration of stay and duration of oxygen requirement. After applying normality tests (Shapiro-Wilk test (p=0.00) and Kolmogorov-Smirnov (p=0.00)), which were significant, the Kruskal-Wallis test was done, which was not significant for duration of stay (H=1.77, p=0.62) and duration of oxygen requirement (H=3.31, p=0.35).

## Discussion

This study was carried out with an overall aim of improving existing data on the contribution of remdesivir in treating patients suffering from COVID-19 to aid future treatment of SARS CoV-2. In the current study, the mean age of patients receiving remdesivir was 57.51 ± 12.98 (30-84) years. In all major studies [11-13,16] conducted, the mean age of patients receiving remdesivir was between 55 and 70 years. It connotes the already proven fact that the requirement for remdesivir is higher in elderly patients. Comorbidity is also considered an important factor. In this study, out of 166 patients, 105 (63.25%) had comorbidity, among which hypertension and diabetes were the most common. Other studies [11,12,15-17] also reported these two comorbidities as the most common in patients requiring remdesivir. It indicates either the role of these comorbidities in worsening disease profiles or the overall increased level of these comorbidities in representing a group of society. The mean duration of stay was 12.80 ± 5.99 (5-30) days. Other studies [13,14,18] reported a duration of stay varying from 10 to 14 days. Although the results of the current study are on par with these studies, Asselstine et al. [19] reported a higher duration of stay of 28 days in remdesivir-treated patients. Since its inception in the COVID-19 pandemic, duration of stay has remained a topic of discussion and paradox in the case of remdesivir use [20]. Due to a lack of control arms, results could not be compared with patients who didn’t receive remdesivir. The mean duration of oxygen requirement was 9.41 ± 7.47 (0-39) days, which is in consonance with another study [14], which observed a 9.42 (8.0-10.8) mean duration of oxygen requirement.

In the COVID-19 pandemic, health care modalities like duration of stay, duration of oxygen requirement, requirement of assisted ventilation, and cure rate are important factors to determine available health care infrastructure, while gender, age, and comorbidity are important risk factors in the progression of severe COVID-19 [21]. Therefore, the association of these health care modalities with gender, age, and comorbidity was explored. Although statistically insignificant, in patients receiving remdesivir, the duration of stay and duration of oxygen requirement were higher in females, while males required more assisted ventilation. Lee et al. [14] published that the duration of stay, duration of oxygen requirement, and intensive care admission were less in males. These findings are partly in unison with the present study. However, instead of the requirement of intensive care admission, the requirement of assisted ventilation is used as a parameter in the present study because the requirement of admission to intensive care can be subjective and may vary according to the treating person, while the requirement of assisted ventilation is devoid of such bias. Previous studies [15,18] have also reported similar findings, with females having more recovery rate. These variations in gender groups among remdesivir-treated patients may be due to differences in community settings in which studies have been conducted.

Age is a crucial factor in the prognosis of COVID-19. In this study, duration of stay and duration of oxygen requirement were both higher in the >60 year age group, and this difference was statistically significant in the case of duration of oxygen requirement and partially significant in the case of duration of stay. The requirement of assisted ventilation was significantly higher in the ≤60 year age group. An equivalent finding is also noted by Richardson et al. [17]. The cure rate was higher in the ≤60 years age group, which is also supported by other studies [15,18-19]. Age-related analysis imparts that even the use of remdesivir is less likely to affect disease prognosis in aged patients.

Comorbidity analysis admits that duration of stay, duration of oxygen requirement, and requirement of assisted ventilation were all higher in comorbid patients, but these differences were not statistically significant. It points that comorbid patients had improved health care modality status after receiving remdesivir. Furthermore, as comorbidity is the most important risk factor, further analysis was done by odds ratio, which showed a strong association of comorbidity with cure rate. It proposes that patients without comorbidity have more chance of getting cured. The present study underlines that as the number of comorbidities increases, it proportionately increases the duration of stay and the duration of oxygen requirement. However, this finding was not statistically significant. Another study conducted by Manudhane et al. [15] also observed that an increase in the number of comorbidities is associated with a decrease in recovery rate. Comorbidity analysis revealed that remdesivir is likely to affect the comorbidity-driven outcome in COVID-19 patients.

Limitations

The current study was restricted to a single tertiary care teaching hospital with a relatively smaller sample size, and results may not be generalized. Further, concomitant treatments like steroids, antibiotics, and immunomodulators may also have affected the outcomes of patients along with remdesivir, which remains out of context in this study. Further studies are warranted to study the effects of remdesivir on clinical outcomes and various health care modalities in COVID-19 patients.

## Conclusions

This is an observational study to analyze the effect of remdesivir on clinical outcomes and various health care modalities. Based on the results of this study, it can be concluded that in remedesivir treated patients, age affects utilization of health care modalities, while gender and comorbidity do not have significant influence. Remdesivir shows better clinical outcomes in female, non-comorbid, and younger patients. Remdesivir can be considered as a beneficial drug in the treatment of COVID-19. This will help in widening the current vision of treatment against COVID-19 infection.
